# A Study on Rejecting Non-Target and Misclassified Motions for Robust Tactile-Sensor-Based Prosthetic Hand Control

**DOI:** 10.3390/s26020721

**Published:** 2026-01-21

**Authors:** Hayato Iwai, Feng Wang

**Affiliations:** 1 Graduate School of Engineering, Maebashi Institute of Technology, Maebashi 371-0816, Japan; h.iwai.mit@gmail.com; 2 Fujitsu Limited, Kawasaki-shi 212-0014, Japan; 3 Faculty of Engineering, Maebashi Institute of Technology, Maebashi 371-0816, Japan

**Keywords:** PVDF sensor, tactile sensor, prosthetic hand control, motion classification, support vector machine, reject option, non-target motions, robustness

## Abstract

Reliable motion classification is essential for practical prosthetic-hand control. Unintended activations caused by ambiguous motions, unknown motions, or non-target body movements can degrade controllability and compromise user safety. Mechanical-sensing approaches are attracting attention as alternatives or complements to surface electromyography, and tactile-sensor-based methods represent one such direction. However, despite extensive studies on prosthetic control, systematic investigations of computationally lightweight motion-rejection strategies remain limited. This study investigates rejection mechanisms to improve the robustness of polyvinylidene fluoride (PVDF) tactile-sensor-based prosthetic control. The proposed approach selectively withholds outputs for misclassified and non-target inputs. We compare three mechanisms: (1) one-class support vector machine (OCSVM) outlier detection, (2) entropy-based rejection using a multilayer perceptron (BPNN-Entropy), and (3) a parameter-free decision-consistency check for one-vs-rest support vector machines (SVMs) that withholds classification when the output sign pattern is inconsistent (one-vs-rest reject option (OvR-RO); proposed). Performance is evaluated for three sources of unintended activation: ambiguous target trials (retrospectively defined), unknown motions excluded from training, and non-target body movements. The results show that OvR-RO achieves a favorable balance between rejection rate and rejection precision for ambiguous motions, while maintaining responsiveness. Overall, explicitly rejecting misclassified and non-target motions is effective for enhancing robustness in tactile-sensor-based prosthetic control.

## 1. Introduction

The practical usability of prosthetic hands strongly depends on the reliability of motion classification. This reliability determines how accurately the system interprets the user’s motor intention. In real-world operation, unintended activations frequently occur due to ambiguous motions, unknown motions, or non-target body movements. Such unintended activations degrade controllability and compromise user safety. These effects are particularly problematic in daily tasks that require stable and repeatable operation. Accordingly, improving the robustness of motion classification has long been recognized as a key requirement for practical prosthetic hand control. Previous reviews have highlighted persistent challenges in electromyography-based control, including instability under real-life conditions [1]. Broader limitations concerning reliability and user acceptance in prosthetic hand systems have also been reported [2]. These observations are consistent with reviews of pattern-recognition-based myoelectric control, which have emphasized issues of robustness and clinical translation in multi-degree-of-freedom prostheses [3,4].

Surface electromyography (sEMG) has long been the dominant sensing modality for prosthetic hand control. However, its long-term stability is affected by changes in skin impedance, perspiration, electrode placement reproducibility, and electrical noise. Several reviews have highlighted that such non-stationarities limit the robustness and long-term usability of myoelectric control systems in daily-life environments [5,6]. These limitations have motivated the exploration of mechanical-sensing approaches that capture the physical consequences of muscle contraction rather than electrical activity. Optical systems that measure wrist contour deformation have shown that surface shape changes contain discriminative information for hand and wrist motions [7]. Muscle-bulging-based approaches have further demonstrated that forearm-surface displacement can be used to classify composite wrist–hand motions [8]. Force-myography (FMG)-based studies have shown that pressure variations derived from muscle swelling yield fast response characteristics. However, FMG implementations have primarily been applied to one-degree-of-freedom control, making their extension to multi-motion discrimination difficult [9]. Together with other mechanical-sensing approaches, these findings suggest that forearm-surface deformation and tension patterns provide useful information for motion classification. At the same time, existing implementations typically require relatively large fixtures or multiple sensing elements, which limits their practicality in compact and wearable prosthetic control systems.

To address these issues, our recent studies have focused on computationally lightweight, skin-conformal tactile sensors based on polyvinylidene fluoride (PVDF) films. These sensors can capture subtle dynamic strain and local deformation on the forearm surface without rigid attachments. We have shown that PVDF-based tactile signals contain discriminative features associated with multiple wrist and hand motions. In offline experiments, we systematically compared the number and combinations of sensing locations, and demonstrated that two sensing locations are sufficient for accurate motion discrimination, with the combination over the flexor carpi radialis (FCR) and extensor carpi radialis longus (ECRL) achieving the highest classification accuracy [10]. Furthermore, using a PVDF configuration with only two sensors, we compared different classifiers and demonstrated that support vector machines enable real-time multi-motion classification with sufficient stability and responsiveness, even when trained with a limited amount of training data [11,12]. These results indicate that PVDF-based tactile sensing provides a compact and reliable foundation for investigating unintended-activation suppression strategies aimed at improving prosthetic control robustness.

In contrast to sEMG, PVDF-based tactile sensing has been reported to exhibit greater robustness against certain real-world disturbances. In particular, prior studies using PVDF-based tactile feature sensing have shown that motion-classification performance is not significantly degraded by perspiration. However, prolonged or severe muscle fatigue can reduce accuracy after extended loading. These findings highlight both the robustness of PVDF-based tactile sensing and the remaining challenges of mechanical-sensing-based prosthetic control [13].

In the broader machine-learning field, rejecting unreliable or out-of-distribution samples has been widely studied under the topic of anomaly detection. A variety of approaches have been proposed, including autoencoders, density-estimation models, and reconstruction-based criteria [14]. Among classical approaches, one-class support vector machine (OCSVM) remains one of the most established novelty-detection methods. This is due to its solid theoretical basis [15] and extensive real-world use [16]. Within the support vector machine (SVM) framework, another important line of research is the reject-option formulation proposed by Fumera and colleagues. They introduced principled decision rules that jointly optimize classification and rejection thresholds [17,18]. Although these works provide theoretical foundations for confidence-controlled SVM decisions, they require explicit threshold tuning and are not inherently optimized for real-time or resource-constrained prosthetic control.

In contrast, work in myoelectric prosthetic control has primarily explored reject options based on neural-network-based uncertainty modeling. Tsuji and colleagues introduced entropy-based rejection for backpropagation neural networks (BPNNs) [19]. This framework was later extended to recurrent log-linearized Gaussian mixture networks (R-LLGMNs) [20]. More recent studies have adopted neural anomaly-detection strategies to reject novel motions in high-density sEMG systems [21]. Such approaches have also been used to suppress unrelated limb movements in low-density systems [22]. While these methods demonstrate the utility of rejection mechanisms, they rely on computationally intensive neural architectures. As a result, they are not always suitable for embedded, real-time prosthetic controllers.

In the realm of mechanical-sensing-based prosthetic control, including optical contour sensing, muscle-bulging deformation sensing, FMG, and tactile sensing, virtually no prior studies have explicitly addressed the rejection of unintended activations. Existing work in this domain has largely focused on improving motion-classification accuracy or demonstrating real-time operation. However, such studies typically do not include mechanisms to detect and suppress ambiguous, unknown, or non-target body movements. As a result, rejection strategies remain a critical yet unexplored challenge for enhancing the reliability of mechanical-sensing-based prosthetic systems.

Motivated by these gaps, the present study systematically investigates computationally lightweight rejection mechanisms suitable for PVDF-based tactile sensing. Building on previous findings that PVDF film sensors enable compact, skin-conformal, and real-time multi-motion discrimination, we evaluate three rejection mechanisms: (1) a one-class SVM for outlier detection (OCSVM), (2) an entropy-based rejection using a backpropagation neural network (BPNN-Entropy), and (3) a decision-consistency check applied to one-vs-rest SVMs, which withholds classification when the output sign pattern is inconsistent (one-vs-rest reject option (OvR-RO); proposed). These methods are examined across three representative sources of unintended activation: ambiguous motions arising within the target classes, unknown motions excluded from training, and non-target body movements. The objective of this study is to determine whether explicit rejection of misclassified or unintended motions can enhance the reliability of tactile-sensor-based prosthetic control while maintaining system responsiveness.

The contributions of this study are summarized as follows: (i) unintended-activation rejection is formulated as a central reliability issue in mechanical-sensing-based prosthetic hand control, an aspect that has received little systematic attention; (ii) a unified experimental framework is established for comparing computationally lightweight reject-option mechanisms under identical conditions using PVDF tactile sensing; (iii) a parameter-free decision-consistency rejection strategy (OvR-RO) is proposed, which introduces negligible computational overhead while preserving real-time operability; and (iv) it is demonstrated that a simple, parameter-free decision-consistency check achieves balanced rejection behavior across ambiguous, unknown, and non-target motions without sacrificing real-time operability.

## 2. Materials and Methods

### 2.1. Participants

A total of nine healthy right-handed adults participated in this study. The participant group consisted of six males (20–60 years) and three females (in their 20s). To reflect physical variability relevant to amputee users—such as differences in musculature strength and subcutaneous fat—the group included individuals with different patterns of upper-arm muscle mass and subcutaneous fat thickness.

For clarity, participants are denoted as A–I, and individual measurement sessions are denoted as A1, A2, B1, B2, C1, C2, D1, E1, F1, G1, H1, and I1. Several participants contributed multiple measurement sessions conducted on different days, resulting in a total of twelve datasets used for analysis. Not all sessions included all experimental conditions: some sessions comprised only the target-motion protocol, whereas others additionally included the unknown-motion and/or non-target body-movement protocols. Participants with prior experience in PVDF-based motion-classification experiments were included together with first-time participants, yielding a dataset that spans a range of task familiarity.

The experiments were conducted with approval from the Ethics Committee of Maebashi Institute of Technology, and written informed consent was obtained from all participants.

### 2.2. Motion Set and Experimental Protocol

The experimental protocol consisted of three motion categories: (1) target motions used for classifier training and standard evaluation, (2) unknown motions excluded from the training set to evaluate rejection performance, and  (3) non-target body movements representing unintended upper-limb activity.

[Trial Allocation]

All twelve session-wise datasets described in Section 2.1 were included in the analyses. Each session contained the motion categories assigned to that dataset, with some sessions covering all three categories (target, unknown, and non-target body movements) and others covering only a subset.
All sessions included the target-motion protocol. Among the twelve sessions, seven sessions additionally included the unknown-motion protocol, and four sessions included the non-target body-movement protocol (elbow flexion). These subsets match the session lists reported in the Results tables for each category.

[Posture and General Measurement Conditions]

**Figure 1:**
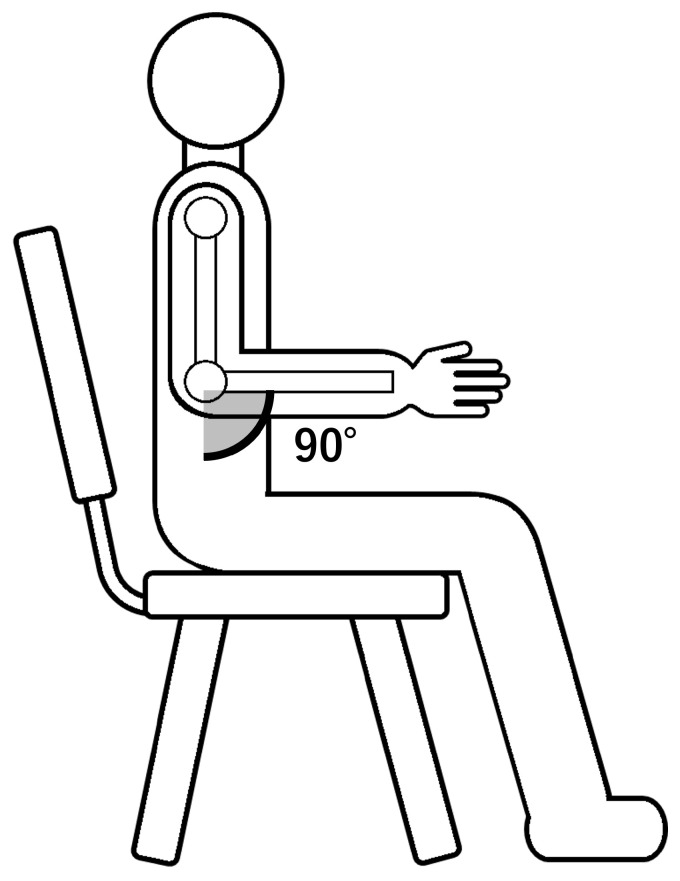
Side view of the general seated posture used for all measurements. Participants were seated upright with the trunk kept vertical. The right upper arm was oriented approximately vertically, with the elbow flexed at approximately 90°. The forearm was positioned forward and kept suspended in mid-air, approximately parallel to the floor, without resting on the desk or armrests.

All measurements were conducted with participants seated upright on a chair. The trunk was kept vertical, and the shoulder angle was maintained constant throughout the experiments. The right elbow was flexed at approximately 90°, and the upper arm was oriented approximately vertical. The forearm was positioned forward and kept suspended in mid-air, without resting on the desk or armrests. The forearm was therefore approximately parallel to the floor. The left arm remained relaxed and was allowed to rest naturally, provided that it did not move during execution of the right-arm motions. The hand posture was maintained in a neutral position: the palm oriented vertically, the ulnar side facing downward, the thumb upward, and the fingers naturally curved with minimal muscle activation. Participants were instructed to avoid moving body parts unrelated to the instructed motion. A schematic side view of the overall posture is shown in Figure 1.

[Cueing and Timing]

All motions were executed in response to an auditory metronome cue occurring every 2 s. Participants initiated each motion immediately after the cue, completed the action within approximately 1 s, and then promptly returned the hand to the neutral position. Unnecessary muscle activation was avoided, and only the force required to complete each motion was permitted.

**Figure 2:**
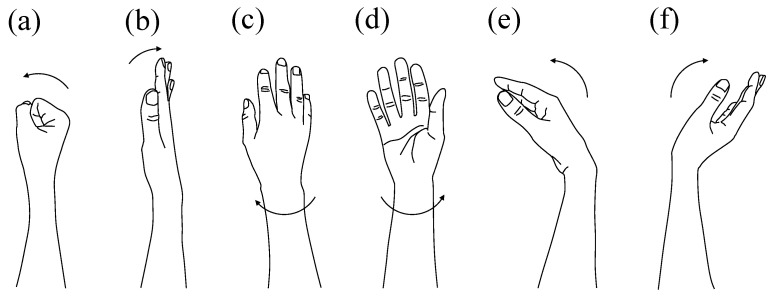
Target wrist–hand motions used in this study. (**a**) Grasp, (**b**) Open, (**c**) Pronation, (**d**) Supination, (**e**) Palmar flexion, (**f**) Dorsiflexion.

(1) Target Motions (including retrospectively identified ambiguous target trials). The target motion set consisted of six wrist–hand actions commonly used in prosthetic control systems. Each motion was repeated 50 times, yielding a total of 300 trials per participant in the target category. A visual reference for the six target motions is provided in Figure 2. These motions were selected based on their frequent use in prosthetic-hand operation and our previous studies.

The six target motions consisted of the following actions:

- Grasp: From the neutral posture, flex the four fingers (excluding the thumb) to close the hand. The thumb lightly contacts the lateral side of the index finger without forceful gripping.
- Open: Extend the fingers and flatten the palm while keeping the wrist relaxed and avoiding wrist extension.
- Pronation: Rotate the forearm so that the palm faces downward (wrist adduction direction).
- Supination: Rotate the forearm so that the dorsal side of the hand faces downward (wrist abduction direction).
- Palmar flexion: Flex the wrist in the palmar direction to the natural limit (approximately 90°) without simultaneous finger flexion.
- Dorsiflexion: Extend the wrist in the dorsal direction to the natural limit (approximately 60°), avoiding finger extension.

These definitions follow the standardized descriptions used in our prior studies and in the doctoral dissertation.

Ambiguity within Target Motions. Ambiguous motions were defined retrospectively as target-motion trials that were misclassified by the corresponding base classifier, because ambiguity cannot be uniquely or objectively labeled a priori and arises from the continuous and overlapping nature of human movements. Accordingly, no dedicated protocol was used to intentionally induce ambiguous activation patterns, in order to avoid arbitrary or biased labeling and to focus on practically ambiguous cases encountered during classification.

**Figure 3:**
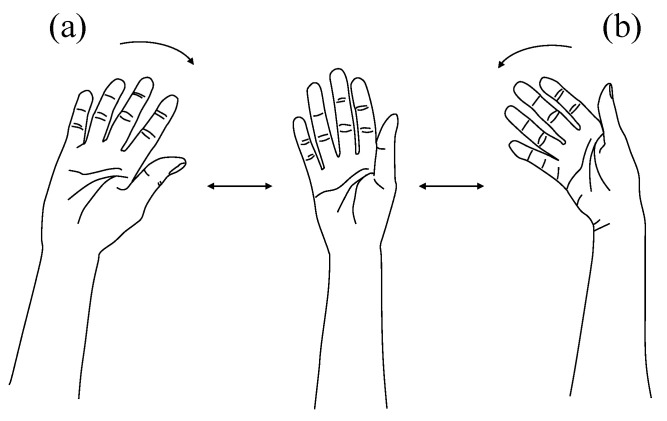
Unknown wrist deviations used to evaluate the rejection performance. (**a**) Radial flexion, (**b**) ulnar flexion.

(2) Unknown Motions (Two Wrist Deviations). Unknown motions consisted of wrist radial flexion and ulnar flexion. These motions were not included in classifier training and were used solely to evaluate rejection performance. A visual reference for the unknown motions is provided in Figure 3.

- Radial flexion: Bend the wrist toward the thumb side.
- Ulnar flexion: Bend the wrist toward the little-finger side.

Each unknown motion was performed 50 times, yielding 100 trials in total for each participant assigned to this condition. Participants were instructed to perform the motions at a natural speed; no specific strength or velocity constraints were imposed.

(3) Non-Target Body Movement (Elbow Flexion). To examine whether the system incorrectly responds to upper-limb movements unrelated to wrist–hand activity, elbow flexion was included as a non-target body movement.

**Figure 4:**
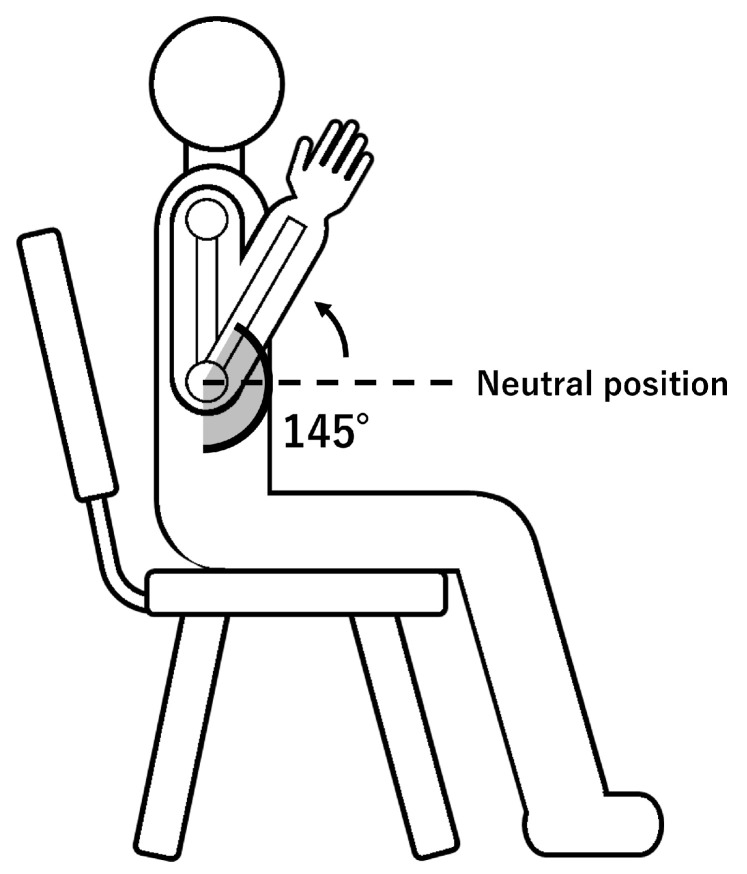
Side view of the posture used for the non-target elbow flexion trial.

Participants started from the same initial posture as the other target motions, with the elbow flexed at approximately 90°, and then further flexed the elbow to a naturally flexed position (approximately 145°), before returning to the initial 90° posture. The shoulder angle, seating posture, and hand–wrist configuration were kept identical to those used for the target motions, so that only the elbow flexion distinguished this non-target movement. A schematic side view of the posture and elbow motion is shown in Figure 4 to avoid ambiguity regarding the experimental configuration. This movement was repeated 50 times.

### 2.3. PVDF-Based Tactile Sensing System

**Figure 5:**
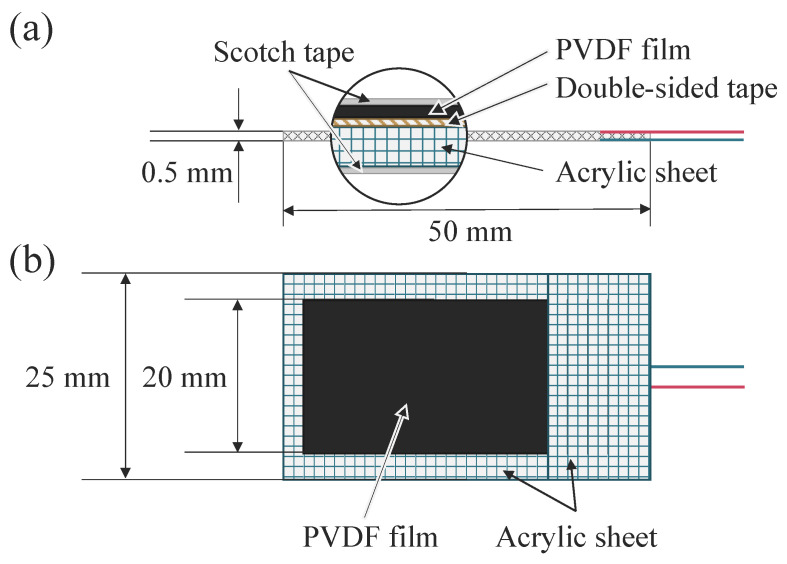
Structure of the polyvinylidene fluoride (PVDF) tactile sensor used in this study. (**a**) Side view showing the cross-sectional structure consisting of the PVDF film, silver electrodes, and the flexible acrylic support layer. (**b**) Top view showing the PVDF film and electrode layout.

The tactile sensing system used in this study was identical to the configuration employed in our previous research on real-time motion classification using forearm-surface tactile behavioral signals [10,11,12]. Two strip-type tactile sensors were constructed from polyvinylidene fluoride (PVDF) film with silver electrodes (200 × 280 × 110 Silver, Measurement Specialities, Inc., Hampton, VA, USA), laminated together with a flexible acrylic sheet that serves as a compliant support layer. The PVDF film was 122 µm thick and provided a flexible, skin-conformal structure capable of generating electric charge in response to local dynamic strain and deformation of the skin surface [11]. The structural design of the tactile sensor is illustrated in Figure 5.

**Figure 6:**
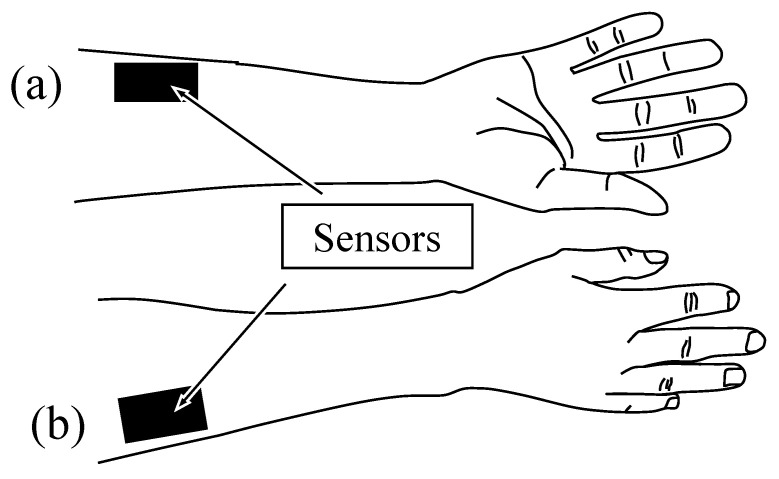
Placement of the PVDF tactile sensors on the proximal volar forearm. (**a**) Sensor positioned above the flexor carpi radialis (FCR). (**b**) Sensor positioned above the extensor carpi radialis longus (ECRL). Both sensors were secured with elastic tape to maintain stable skin contact during motion execution.

Each PVDF sensor was attached to the proximal volar forearm above the FCR and ECRL muscle bellies, enabling acquisition of deformation patterns produced by muscle contraction and relaxation. The placement of the two sensors on the forearm is shown in Figure 6. The sensors were secured to the skin using elastic tape and covered with elastic supporters to maintain stable contact during wrist and hand movements.

The electric charge generated by the PVDF film was converted into a voltage signal using a charge amplifier (4001B, Showa Sokki Corporation, Tokyo, Japan). Prior to data collection, the amplifier gain for each channel was adjusted so that the output voltage range remained within ±5 V, matching the dynamic range of the subsequent A/D conversion stage. The amplified signals were then low-pass filtered using a programmable filter (3628, NF Corporation, Yokohama, Japan) configured as an eighth-order Butterworth filter with a cutoff frequency of 40.0 Hz.

Filtered signals were digitized using a multi-channel data recorder (EZ7510, NF Corporation, Yokohama, Japan) at a sampling frequency of 1000 Hz with 16-bit resolution. This configuration was selected to maintain consistency with previously validated experimental setups and to preserve dynamic tactile components essential for motion classification.

The experimental system used in the present study was implemented as a laboratory-scale setup, and the overall configuration is relatively large. However, as demonstrated in our previous work [12], the signal-conditioning and processing components can be readily miniaturized using a compact signal-processing board of a size comparable to an Arduino Uno single-board controller kit, together with embedded platforms such as Arduino or Raspberry Pi. Furthermore, through surface-mount implementation and further integration of the electronic components, the entire sensing and processing system can be substantially downsized and embedded into a powered prosthetic hand without fundamental technical difficulty. Accordingly, the present experimental configuration should be regarded as an evaluation platform, rather than a limitation on practical deployment.

**Figure 7:**
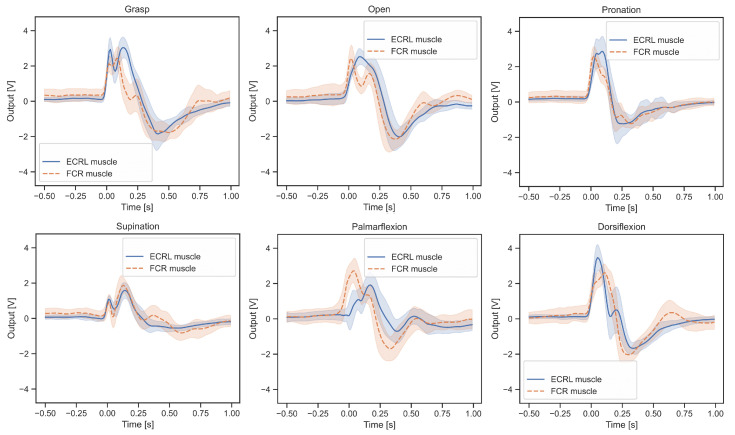
Representative averaged voltage waveforms measured from the two PVDF tactile sensors. Solid line: FCR channel; dashed line: ECRL channel. Shaded regions denote the standard deviation across 50 trials.

Representative voltage waveforms obtained after charge amplification are shown in Figure 7. For each of the six target motions, 50 trials were time-aligned to the moment of motion detection and averaged. The solid curves represent the FCR-channel sensor, the dashed curves represent the ECRL-channel sensor, and the shaded regions indicate one standard deviation across trials. These examples illustrate the characteristic deformation-induced responses captured by the PVDF tactile sensors and provide the basis for subsequent signal processing and feature extraction.

### 2.4. Signal Processing and Feature Extraction

**Figure 8:**
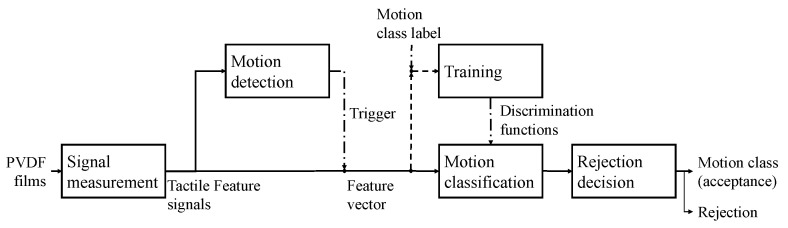
Overview of the signal-processing pipeline used in this study. PVDF films measure forearm-surface deformation, followed by event-driven motion detection. Detected segments are converted into feature vectors and classified using discrimination functions obtained during training. The classification output is subsequently evaluated by a rejection decision module, which determines whether the motion is accepted as a target motion class or rejected as unintended.

All analyses in this study were conducted offline using the PVDF tactile signals obtained as described in Section 2.3. The overall signal-processing pipeline—including measurement, event-driven detection, feature construction, and classification—is summarized in Figure 8. The signal-processing and feature-extraction pipeline followed the configuration used in our previous real-time implementation [10], ensuring consistency between the present offline analysis and the prior online system. The same feature vectors were used for all classifiers and rejection mechanisms to ensure a fair comparison.

#### 2.4.1. Digital Signal Processing

The analog tactile signals were conditioned using the charge amplifier and eighth-order low-pass filter described in Section 2.3, and were then digitized at 1000 Hz with 16-bit resolution. To suppress baseline drift and low-frequency offset components, each channel was processed using a first-order high-pass filter with a cutoff frequency of 0.5 Hz. In addition, a second-order notch filter centered at 50 Hz with a quality factor of Q=25 was applied to attenuate power-line interference. These digital filters were applied to all channels prior to downsampling.

After filtering, the signals were downsampled to 100 Hz by averaging every 10 consecutive samples. This downsampling step reduced computational load while retaining the dynamic deformation components relevant for wrist–hand motion classification.

#### 2.4.2. Event-Driven Motion Detection

Following our previous real-time system [10], an event-driven motion detection scheme was adopted. For each participant and sensor channel, a detection threshold $\theta_{i}$ was determined from resting segments recorded before the experiment. The motion detection time $t_{d}$ was defined as the first time index at which the absolute value of at least one channel exceeded its threshold:

(1) $$|x_{i}\left[n\right]|>\theta_{i}.$$

This detection moment was treated as t=0 for subsequent feature extraction. The same detection rule was applied to target motions, unknown motions, and non-target body movements in order to emulate the behavior of an online prosthetic controller.

#### 2.4.3. Segment Extraction and Feature Vector Construction

A fixed-length segment centered on the detection moment was extracted for feature construction. Consistent with the real-time system described in [10], a 200 ms window was used, spanning from 100 ms before to 100 ms after the detection moment:

(2) t∈[−100ms,100ms].

At a sampling frequency of 100 Hz, this corresponds to 20 samples per channel. For the *i*-th PVDF sensor, the extracted sequence is as follows:

(3) $$x_{i}={\left[x_{i}\left[n_{d-10}\right],\;x_{i}\left[n_{d-9}\right],\;\ldots ,\;x_{i}\left[n_{d+9}\right]\right]}^{⊤}\in R^{20},$$

where $n_{d}$ denotes the discrete-time index corresponding to the detection time.

The feature vector was constructed by concatenating the samples of all *l* channels:

(4) $$z={\left[{x_{1}}^{⊤},\;{x_{2}}^{⊤},\;\ldots ,\;{x_{l}}^{⊤}\right]}^{⊤}\in R^{20l}.$$

With two PVDF sensors (l=2), the resulting feature vector had a dimensionality of 40.

The same signal-processing and feature-extraction procedure was applied to all motion categories. Target-motion segments were used for training and standard evaluation. Segments from unknown motions (radial and ulnar flexion) and non-target body movements (elbow flexion) were excluded from training and used only during testing to evaluate rejection performance.

### 2.5. Classifier Implementation and Rejection Mechanisms

Three rejection mechanisms were compared in this study using the feature representation described in Section 2.4. All classifiers were implemented using identical training–test partitions for each participant. Hyperparameters were determined in preliminary analyses based on receiver operating characteristic (ROC) curves computed across all participants. For each rejection mechanism, the operating point was selected as the parameter setting that minimized the Euclidean distance to the ideal point $\left({\mathrm{RR}}_{\mathrm{target}},\;{\mathrm{RR}}_{\mathrm{unintended}}\right)=\left(0,\;1\right)$, where ${\mathrm{RR}}_{\mathrm{target}}$ denotes the rejection rate for target motions and ${\mathrm{RR}}_{\mathrm{unintended}}$ denotes the rejection rate for unintended inputs (ambiguous, unknown, and non-target motions). The selected parameters were then fixed and applied uniformly across all participants and motion classes, without participant-specific tuning. This design ensured a fair comparison among rejection mechanisms and maintained consistency with our prior real-time implementation.

#### 2.5.1. Baseline Multi-Class Classifier: One-vs-Rest SVM (Baseline SVM)

A multi-class SVM with a one-vs-rest (OvR) architecture served as the baseline classifier (Baseline SVM) for the six target motions. A radial basis function (RBF) kernel was used with fixed hyperparameters: regularization parameter C=16 and kernel width γ=0.0625. These values were determined through the preliminary ROC-based analyses and subsequently applied identically across all participants and target classes. For an input feature vector z, the classifier outputs six decision-function values:

(5) $$f_{k}\left(z\right),\;k=1,\ldots ,6,$$

and the motion corresponding to the maximum value is selected as the predicted label.

For all SVM-based methods, ambiguous motions were defined retrospectively as target-motion trials that were misclassified by the Baseline SVM. These trials naturally arose from biomechanically similar wrist–hand activations and were not intentionally induced during the experiment.

#### 2.5.2. Rejection Mechanism 1: OCSVM (One-Class SVM)

The first rejection mechanism employed a one-class SVM (OCSVM) trained exclusively on target-motion feature vectors. OCSVM is a classical novelty-detection method with strong theoretical grounding [15] and widespread use in anomaly-detection applications across various sensing domains [16]. In this study, it served as a representative non-neural anomaly-detection baseline suitable for comparison with the proposed SVM-based reject option.

An RBF kernel was used. The hyperparameter ν was fixed to 0.1, controlling the expected fraction of outliers. The kernel parameter γ was set using the scale heuristic, defined as γ=1/(d·Var(z)), where d=40 is the feature dimension. Both parameters were selected based on the preliminary ROC analyses and then fixed across all participants for consistency.

For a given input z, the OCSVM computes a signed decision value Δ(z). A sample was accepted when the following was true:

(6) Δ(z)>0,

Otherwise, it was rejected. Motions not represented in the training data—such as unknown wrist motions and non-target body movements—typically fell outside the learned decision boundary and therefore yielded negative decision values.

#### 2.5.3. Rejection Mechanism 2: BPNN-Entropy

This mechanism followed the classical entropy-based rejection framework originally introduced by Tsuji et al. [19]. A two-layer multilayer perceptron (MLP) was used, consisting of a 40-dimensional input layer, one hidden layer with 25 units and a tanh activation function, and a softmax output layer producing class probabilities $p_{k}\left(z\right)$ for the six target motions.

The network parameters were trained to minimize the multiclass cross-entropy loss using the Adam optimizer [23]. The update rule for the weight vector θ followed the standard Adam formulation with parameters $\beta_{1}=0.9$, $\beta_{2}=0.999$, $\epsilon =10^{-8}$, and learning rate α=0.001, consistent with our previous work [11]. The initial moment estimates were set to zero, and the initial weights were drawn from a uniform distribution in [0, 1.0). Training was performed for 1000 epochs using batch learning.

Uncertainty was quantified using Shannon entropy,

(7) $$H\left(z\right)=-\sum_{k=1}^{6}p_{k}\left(z\right)\log p_{k}\left(z\right),$$

and an input was rejected when H(z)>τ. The threshold was fixed at τ=0.7, selected through the preliminary ROC-based analyses and applied uniformly across all participants and classes. Ambiguous motions for this mechanism were defined as target-motion trials that were misclassified by the trained BPNN classifier.

#### 2.5.4. Rejection Mechanism 3 (Proposed): OvR-RO (Decision-Consistency SVM)

The proposed rejection mechanism (OvR-RO) extended the Baseline SVM by examining the sign consistency of its one-vs-rest decision-function outputs. Within the SVM literature, a closely related line of research is the reject-option formulation proposed by Fumera and colleagues [17,18], which embeds classification and rejection thresholds into a unified SVM decision rule. While these approaches provide a principled framework for confidence-controlled SVM decisions, they typically require explicit threshold optimization and additional hyperparameter tuning.

In contrast, the present method adopted a computationally lightweight, parameter-free strategy based solely on the sign patterns of the OvR decision functions. Each decision value $f_{k}\left(z\right)$ indicates whether class *k* is supported ($f_{k}>0$) or rejected ($f_{k}<0$). Ambiguous or non-target inputs tended to produce inconsistent sign patterns, such as the following:

1. Multiple classes with positive signs;
2. No classes with positive signs.

Both conditions indicate that the classifier cannot make a reliable decision. Therefore, an input is rejected whenever the number of positive signs deviates from exactly one:

(8) $$\#\{k:f_{k}\left(z\right)>0\}\neq 1\;\Rightarrow \;\mathrm{rejected}.$$

Table 1 summarizes the idealized sign patterns for representative input types. Although unknown or non-target inputs do not always produce strictly all-negative patterns in practice, they generally fail to match any single target class and therefore yield zero or multiple positive signs, leading OvR-RO to reject them.

Because OvR-RO required no additional parameters and relied only on a simple consistency check applied to an existing OvR-SVM classifier, it introduced virtually no computational overhead and required no retraining. These properties made it particularly suitable for resource-limited embedded prosthetic controllers.

### 2.6. Evaluation Metrics and Statistical Analysis

The performance of each rejection mechanism was evaluated using the motion categories described in Section 2.2: target motions, ambiguous motions, unknown motions, and non-target body movements. All evaluations were performed separately for each session (dataset).

All hyperparameters for the SVM, OCSVM, and BPNN-based models were fixed across participants and motion classes, and no participant-specific or class-specific tuning was performed.

#### 2.6.1. Evaluation Metrics

Three primary aspects of performance were assessed: (1) classification accuracy for target motions, (2) rejection rate for each motion category, and (3) rejection precision.

(1) Classification Accuracy for Target Motions.

For trials belonging to the six target motions and not rejected by the rejection mechanism, classification accuracy was computed as follows:

(9) $$\mathrm{Accuracy}=\frac{\mathrm{Number}\;\mathrm{of}\;\mathrm{correctly}\;\mathrm{classified}\;\mathrm{target}\;\mathrm{trials}}{\mathrm{Number}\;\mathrm{of}\;\mathrm{accepted}\;\mathrm{target}\;\mathrm{trials}}.$$

This metric was used to evaluate whether the rejection mechanisms improved classification performance by suppressing misclassified target-motion trials.

(2) Rejection Rate.

Rejection rate quantifies how frequently the classifier withholds its output for a given motion category. For each category (target, ambiguous, unknown, and non-target body movement), the rejection rate was computed as follows:

(10) $$\mathrm{Rejection}\;\mathrm{Rate}=\frac{\mathrm{Number}\;\mathrm{of}\;\mathrm{rejected}\;\mathrm{trials}}{\mathrm{Total}\;\mathrm{trials}\;\mathrm{in}\;\mathrm{the}\;\mathrm{category}}.$$

A low rejection rate is desirable for target and ambiguous motions, since these represent intended wrist–hand actions. In contrast, a high rejection rate is desirable for unknown motions and non-target body movements, as these inputs should ideally be suppressed to prevent unintended activations.

(3) Rejection Precision.

Rejection precision measures the selectivity of the rejection mechanism, that is, how reliably rejected trials correspond to unintended inputs:

(11) $$\mathrm{Rejection}\;\mathrm{Precision}=\frac{\mathrm{Number}\;\mathrm{of}\;\mathrm{rejected}\;\mathrm{unintended-motion}\;\mathrm{trials}}{\mathrm{Total}\;\mathrm{number}\;\mathrm{of}\;\mathrm{rejected}\;\mathrm{trials}}.$$

High rejection precision indicates that the mechanism avoids unnecessarily rejecting valid target motions.

#### 2.6.2. Statistical Analysis

All metrics were computed on a per-session (dataset) basis, and paired *t*-tests were applied to these session-wise values. For multiple pairwise comparisons, the significance level was adjusted using the Bonferroni correction:

(12) $$\alpha_{\mathrm{adj}}=\frac{0.05}{N_{\mathrm{comp}}},$$

where $N_{\mathrm{comp}}$ denotes the number of pairwise comparisons.

For classification accuracy, the four systems (Baseline SVM, BPNN-Entropy, OCSVM, and OvR-RO) were compared, yielding $N_{\mathrm{comp}}=6$.

For rejection-related metrics (rejection rate and rejection precision), only the three rejection mechanisms (BPNN-Entropy, OCSVM, and OvR-RO) were compared, because the Baseline SVM has no reject option. Accordingly, $N_{\mathrm{comp}}=3$ was used for Bonferroni correction.

## 3. Results

For readability, we report (i) target-motion accuracy computed on accepted target trials (i.e., trials not withheld by the rejection mechanism), (ii) rejection rate computed on all trials within each category, and (iii) rejection precision, defined as the fraction of unintended trials among all rejected trials.

This section presents the evaluation results of the four classification systems: (1) the Baseline SVM (one-vs-rest SVM without rejection), (2) BPNN-Entropy, (3) OCSVM, and (4) the proposed method based on decision-consistency checking of one-vs-rest SVM outputs (OvR-RO). All analyses were conducted on a session-wise basis, treating the twelve datasets (A1–I1, A2–C2) introduced in Section 2.1 as independent evaluation units.

We focus on three sources of unintended activation, as defined in Section 2.2: (1) ambiguous motions that arise within the target-motion set, (2) unknown motions excluded from training, and (3) non-target body movements (elbow flexion). For each category, we evaluated recognition accuracy (where applicable), rejection rate, and rejection precision as defined in Section 2.6.

### 3.1. Performance on Ambiguous Motions

#### 3.1.1. Recognition Accuracy

Table 2 summarizes the recognition accuracy for target motions for each session and each method. When restricted to the target-motion datasets, the Baseline SVM achieved a mean accuracy of 84.5%, while BPNN-Entropy and OvR-RO yielded higher mean accuracies of 93.0% and 90.0%, respectively. OCSVM showed a mean accuracy of 84.7%, which was comparable to the Baseline SVM.

A paired *t*-test (paired across the twelve sessions) was conducted to compare methods. Compared with the Baseline SVM, BPNN-Entropy showed a significant improvement (mean difference = 8.5 percentage points, p=0.00041), and OvR-RO also significantly improved recognition accuracy (mean difference = 5.5 percentage points, p=0.00234). In contrast, OCSVM did not differ significantly from the Baseline SVM (mean difference = 0.2 percentage points, p=0.724). When comparing the three rejection mechanisms with each other, BPNN-Entropy tended to yield higher accuracy than OvR-RO (mean difference = 3.0 percentage points, p=0.18), although this difference did not reach significance under the Bonferroni-corrected threshold. BPNN-Entropy also significantly outperformed OCSVM (mean difference = 8.3 percentage points, p=0.00032), and OvR-RO significantly outperformed OCSVM (mean difference = 5.3 percentage points, p=0.00600).

These results indicate that both BPNN-Entropy and OvR-RO improve recognition performance by selectively rejecting ambiguous motions within the target-motion set, thereby reducing misclassifications and increasing accuracy. In contrast, OCSVM does not provide a clear advantage in terms of recognition accuracy.

#### 3.1.2. Rejection Rate

Table 3 presents the rejection rate for target motions, which include both unambiguous and ambiguous target trials. Since ambiguous motions are a subset of the target-motion set, this metric reflects how frequently each rejection mechanism withholds output for intended wrist–hand actions.

BPNN-Entropy exhibited the highest mean rejection rate for target motions (32.3%), followed by OCSVM (28.7%) and OvR-RO (14.5%). Thus, BPNN-Entropy and OCSVM rejected a larger proportion of target-motion trials, indicating more conservative behavior rather than higher selectivity. Because excessive rejection of valid target trials reduces responsiveness, lower and more selective rejection is generally preferable for practical usability.

A paired *t*-test revealed that the difference in mean rejection rate between BPNN-Entropy and OCSVM (mean difference = 3.6 percentage points, p=0.034) was not significant under the Bonferroni-corrected threshold. In contrast, BPNN-Entropy showed a significantly higher rejection rate than OvR-RO (mean difference = 17.8 percentage points, p=0.001). The difference between OvR-RO and OCSVM (mean difference = −14.2 percentage points, p=0.017) did not reach the corrected significance level.

#### 3.1.3. Rejection Precision

Table 4 shows the rejection precision for ambiguous motions, defined as the proportion of ambiguous trials among all rejected trials. On average, OvR-RO achieved the highest rejection precision (52.5%), followed by BPNN-Entropy (37.3%) and OCSVM (16.2%). In other words, when OvR-RO withheld an output, the rejected trial was more likely to correspond to an ambiguous target-motion trial.

Paired *t*-tests indicated that BPNN-Entropy achieved significantly higher rejection precision than OCSVM (mean difference = 21.1 percentage points, p=0.004). OvR-RO significantly outperformed OCSVM (mean difference = 38.8 percentage points, p=0.001). Although BPNN-Entropy tended to yield lower precision than OvR-RO (mean difference = −17.7 percentage points, p=0.048), this difference did not reach significance after Bonferroni correction.

These results suggest that BPNN-Entropy achieves high recognition accuracy partly by rejecting a large number of trials, whereas OvR-RO selectively focuses its rejection on truly ambiguous cases, thus achieving higher precision while maintaining accuracy.

### 3.2. Rejection of Unknown Motions

Unknown wrist motions (radial and ulnar flexion), which were excluded from the training set, were used to evaluate how effectively each method rejects motions that are outside the intended control repertoire. The Baseline SVM does not provide a rejection mechanism and therefore yielded a rejection rate of 0% for unknown motions.

Table 5 presents the rejection rates for unknown motions. BPNN-Entropy achieved the highest mean rejection rate (71.7%), followed by OCSVM (44.9%) and OvR-RO (42.7%). A paired *t*-test revealed that BPNN-Entropy significantly outperformed OCSVM (mean difference = 26.8 percentage points, p=0.009) and OvR-RO (mean difference = 29.0 percentage points, p=0.004). The difference between OCSVM and OvR-RO (mean difference = −2.2 percentage points, p=0.28) was not significant.

These results indicate that BPNN-Entropy provides the strongest suppression of unknown motions, while OCSVM and OvR-RO exhibit moderate and comparable rejection capabilities for this category.

### 3.3. Rejection of Non-Target Body Movements

Elbow flexion was used as a representative non-target body movement unrelated to wrist–hand activity. As in the case of unknown motions, the Baseline SVM did not reject any trials in this category.

Table 6 summarizes the rejection rates for non-target body movements. OCSVM and BPNN-Entropy exhibited similarly high mean rejection rates (73.5% and 69.0%, respectively), whereas OvR-RO showed a lower mean rejection rate of 35.5%.

Paired *t*-tests indicated that the difference between BPNN-Entropy and OCSVM (mean difference = −4.5 percentage points, p=0.230) was not significant. In contrast, both BPNN-Entropy and OCSVM showed significantly higher rejection rates than OvR-RO: BPNN-Entropy vs. OvR-RO (mean difference = 33.5 percentage points, p=0.006) and OCSVM vs. OvR-RO (mean difference = 38.0 percentage points, p=0.004).

These results suggest that OCSVM and BPNN-Entropy are more effective in suppressing large non-target body movements, while OvR-RO is comparatively conservative in this category, prioritizing selective rejection of ambiguous trials.

### 3.4. Summary of Rejection Behavior

The three rejection mechanisms exhibit clearly distinct rejection characteristics.

- BPNN-Entropy: shows broadly conservative behavior, producing the highest rejection rates for target motions and unknown motions, and similarly high rejection rates for non-target body movements. Although this reduces some unintended activations, the high rejection of valid target trials limits usability.
- OCSVM: demonstrates moderate rejection behavior overall, with target- and unknown-motion rejection rates generally lower than or comparable to those of BPNN-Entropy, but the highest rejection rate for non-target body movements. Its rejection precision for ambiguous (target-subset) trials is relatively low, indicating limited selectivity.
- OvR-RO (proposed method): produces the lowest rejection rate for target motions while achieving the highest rejection precision for ambiguous (misclassified) target trials. It avoids excessive suppression of valid target outputs and maintains competitive recognition accuracy, rejects unknown motions with a strength comparable to OCSVM, and rejects non-target body movements less aggressively than BPNN-Entropy and OCSVM.

These trade-offs indicate that BPNN-Entropy is suitable when strong suppression of any unintended activation is prioritized, whereas OvR-RO is more appropriate when selective rejection of truly ambiguous motions is required without overly sacrificing responsiveness to intended target motions. A more detailed interpretation of these behaviors and their implications for prosthetic hand control is provided in Section 4.

## 4. Discussion

This study systematically compared three rejection mechanisms in the context of improving the reliability of tactile-sensor-based prosthetic hand control: entropy-based rejection using a multilayer perceptron (BPNN-Entropy), outlier detection using a one-class SVM (OCSVM), and the proposed one-vs-rest sign-consistency check (OvR-RO). The results obtained for target motions (including ambiguous trials), unknown motions, and non-target body movements revealed distinct characteristics related to the trade-off between safety and usability in real-time motion classification.

### 4.1. Interpretation of Performance on Ambiguous Motions

Ambiguous motions are practically critical because they arise when target movements are performed with insufficient or unstable muscle activation. These motions occur within the target-motion set and therefore directly affect both recognition accuracy and user operability. Accordingly, classifier behavior was evaluated in terms of improvements in recognition accuracy, the frequency with which target motions were rejected, and the selectivity with which ambiguous trials were withheld (i.e., rejection precision). Because excessive rejection of intended motions directly reduces operability, any improvement in accuracy and precision must be achieved without substantially sacrificing responsiveness.

The proposed OvR-RO achieved the lowest rejection rate for the target-motion set (14.5%). At the same time, it maintained competitive recognition accuracy (90.0%) and achieved the highest rejection precision for ambiguous trials (52.5%). In contrast, BPNN-Entropy and OCSVM exhibited substantially higher rejection rates (32.3% and 28.7%, respectively). These results indicate that these methods withheld a large number of borderline or partially ambiguous target-motion trials.

From a practical perspective, rejection rates close to 30%, as observed for BPNN-Entropy, are generally incompatible with stable prosthetic operation. Under such conditions, users would frequently experience rejected attempts during natural variations in muscle activation. Although OCSVM exhibited a slightly lower rejection rate (28.7%), more than one in four intended motions would still be withheld. Such behavior would noticeably reduce responsiveness and intuitiveness in daily use. In contrast, the much lower rejection rate of OvR-RO provides a more practical balance. It suppresses unintended activations while avoiding substantial interference with intended control.

### 4.2. Interpretation of Performance on Unknown Motions and Body Movements

For unknown motions and non-target body movements, BPNN-Entropy and OCSVM recorded high rejection rates (71.7% and 44.9% for unknown motions; 69.0% and 73.5% for body movements, respectively). At first glance, these results may appear to indicate superior robustness. However, these values largely reflect the intrinsically conservative nature of the two methods: both employ decision strategies that reject a broad range of input patterns when classification confidence is low or when the sample lies far from the learned manifold. The same tendency is also observed for target motions, where BPNN-Entropy and OCSVM reject a larger proportion of trials than OvR-RO.

In contrast, while OvR-RO also rejected a substantial proportion of unknown trials (42.7%), its behavior was more selective and moderate. OvR-RO triggers rejection only when the one-vs-rest decision boundaries exhibit contradictory evidence, thereby preventing unnecessary over-rejection. This results in a more balanced rejection profile, which is advantageous for systems where both safety and operability must be simultaneously satisfied.

### 4.3. Unified Comparison Across All Categories

To facilitate an integrated evaluation, Table 7 summarizes classification accuracy, rejection rate, and rejection precision across all motion categories considered in this study.

### 4.4. Interpretation of Rejection Behaviors

It is important to emphasize that the high rejection rates observed for BPNN-Entropy and OCSVM under the unknown-motion and body-movement conditions do not directly indicate superior discriminative ability. Instead, these values reflect the use of highly conservative rejection criteria.

Both methods reject a large proportion of borderline or partially ambiguous trials. As a consequence, the rejection rate is increased as a byproduct of over-conservativeness. The same tendency is evident for target motions. In this case, BPNN-Entropy and OCSVM reject more intended trials than the proposed OvR-RO.

This behavior represents a clear trade-off between safety and usability. High rejection rates improve suppression of unintended activations, but they simultaneously reduce responsiveness to intended inputs.

In contrast, the proposed OvR-RO performs selective rejection only when the one-vs-rest decision outputs exhibit inconsistent evidence. As summarized in Table 7, OvR-RO achieves the lowest rejection rate for target motions, including ambiguous trials, which constitute the most practically critical category. At the same time, it maintains competitive robustness against unknown motions and non-target body movements.

This selective and moderate level of conservativeness offers practical advantages for real-time prosthetic control. In such systems, excessive conservativeness can directly hinder operability.

### 4.5. Implications for Real-Time Prosthetic Control

Overall, these findings demonstrate that rejection mechanisms should be evaluated not only by their ability to suppress unintended activations, but also by their selectivity. The proposed OvR-RO achieves a favorable balance. It is sufficiently conservative to prevent unintended activations, yet it avoids excessive rejection of intended movements.

An additional practical advantage of OvR-RO is its computational efficiency. The method is built directly on a one-vs-rest SVM, which has already been validated for fast and stable real-time operation in our previous studies. The rejection process consists solely of a sign-consistency check. As a result, no retraining is required, and only minimal computational overhead is introduced.

This simplicity ensures that the overall processing pipeline remains computationally lightweight. Consequently, OvR-RO is well suited for embedded prosthetic controllers with limited computational resources.

Given these characteristics, OvR-RO aligns well with the requirements of real-time prosthetic hand control. In such applications, responsiveness, stability, and low computational cost must be simultaneously satisfied under the constraints of wearable hardware.

It should be noted that the present evaluation was conducted under controlled laboratory conditions with fixed sensor placement. Common real-world variations, such as perspiration, fatigue progression, and measurement noise, were not systematically examined in this paper. However, our prior studies on PVDF-based tactile sensing have reported that motion-classification performance is largely unaffected by perspiration and mild muscle fatigue. Only prolonged or severe fatigue has been shown to cause noticeable degradation after extended loading.

Therefore, the proposed system is not expected to be fundamentally compromised by these factors in typical daily use. Nevertheless, their potential influence on rejection behavior itself remains an important topic for future investigation [13].

## 5. Conclusions

This study investigated rejection mechanisms for enhancing the reliability of prosthetic hand control based on PVDF tactile sensing. Using a unified processing pipeline, three reject-option strategies were systematically compared: entropy-based rejection using a backpropagation neural network (BPNN-Entropy), outlier detection with a one-class SVM (OCSVM), and a decision-consistency check applied to one-vs-rest SVMs (OvR-RO, proposed). This proposed method is computationally lightweight. The comparison covered three major sources of unintended activation: ambiguous motions within the target set, unknown motions excluded from training, and non-target body movements. Through this analysis, the trade-offs among recognition accuracy, rejection rate, and rejection precision were clarified for each mechanism.

For ambiguous motions, all rejection mechanisms improved recognition accuracy relative to the Baseline SVM without rejection. BPNN-Entropy achieved the highest accuracy. However, it rejected more than 30% of target-motion trials, indicating overly conservative behavior that would substantially reduce responsiveness in practical prosthetic use. In contrast, the proposed OvR-RO combined high recognition accuracy with a lower rejection rate and the highest rejection precision. It selectively suppressed only the most uncertain trials while maintaining operability.

For unknown motions and non-target body movements, BPNN-Entropy and OCSVM exhibited strong suppression. This behavior reflects their suitability for rejecting out-of-distribution inputs. At the same time, their tendency to reject a considerable portion of target motions highlights the need for careful trade-offs between safety and usability. OvR-RO demonstrated more moderate suppression of unknown and non-target movements. Importantly, it required no additional training or hyperparameter tuning and incurred negligible computational cost. These properties make OvR-RO well suited for embedded, real-time prosthetic controllers.

A key contribution of this work is that it addresses an aspect that has received very limited systematic attention in conventional prosthetic-hand research. This includes studies based on myoelectric signals as well as those employing mechanical or tactile sensing. Specifically, this study focuses on the explicit rejection of ambiguous, unknown, and non-target motions. While prior work has predominantly emphasized classification accuracy or real-time operation, systematic evaluation of reject-option strategies has been rare. By comparing multiple rejection mechanisms under consistent experimental conditions, the present study provides a unified basis for designing robust decision-making pipelines for compact PVDF-based sensing systems.

Overall, the findings demonstrate that explicitly rejecting misclassified or unintended motions is an effective strategy for improving the reliability of tactile-sensor-based prosthetic control. Among the mechanisms examined, the proposed decision-consistency framework offers a practical balance between robustness and responsiveness. It provides high interpretability, requires no additional training, and introduces minimal computational overhead. Importantly, the primary conclusions of this study concern the relative and qualitative behavior of different rejection strategies, rather than the absolute numerical performance obtained for a specific set of motions.

Future work will expand the scope of validation by increasing the number and diversity of participants, including amputee users. This will allow inter-participant variability and clinical applicability to be assessed more comprehensively. In addition, a broader range of unknown and non-target motions, including more diverse real-world movements, will be incorporated. This will enable examination of whether the qualitative trends observed in the relative behavior of rejection mechanisms remain consistent beyond the representative motion types considered in this initial study.

Further investigation will also address practical deployment aspects. These include real-time closed-loop control experiments and quantitative evaluation of processing latency under embedded hardware constraints. Although prior studies have indicated that PVDF-based tactile sensing is largely robust to perspiration and mild muscle fatigue, future work will explicitly examine how such real-world factors influence rejection behavior itself. This is particularly important for prolonged or continuous-use scenarios.

Finally, hybrid rejection architectures that combine conservative anomaly detection with selective ambiguity handling will be explored. Such approaches may further improve the balance among safety, robustness, and responsiveness in practical prosthetic hand control.

## Figures and Tables

**Table 1:** Conceptual behavior of OvR-RO. Sign patterns show idealized one-vs-rest decision-function signs. For brevity, “+−−−−−” represents the six-class sign pattern.

Input Type	Signs ($f_{1}\ldots f_{6}$)	#Pos	Output
Target (Grasp)	+−−−−−	1	Accept
Target (Dorsiflex.)	−−−−−+	1	Accept
Ambiguous (G–P midpoint)	+−+−−−	2	Reject
Ambiguous (Weak)	−−−−−−	0	Reject
Unknown	−−−−−−	0	Reject
Body movement	−−−−−−	0	Reject

**Table 2:** Recognition accuracy for target motions across sessions (A1–I1, A2–C2).

Session	Baseline SVM	BPNN-Entropy	OCSVM	OvR-RO
A1	99.9	100.0	100.0	99.9
A2	90.9	96.0	89.0	93.1
B1	91.8	97.6	93.5	95.7
B2	86.1	92.3	85.3	90.4
C1	82.0	92.6	83.4	89.2
C2	80.8	93.3	77.0	85.1
D1	90.1	94.1	92.1	92.9
E1	91.3	97.6	92.8	94.2
F1	85.2	93.6	87.6	90.7
G1	52.7	74.9	52.0	67.5
H1	70.3	85.3	68.2	86.0
I1	92.8	98.4	95.6	95.5
Mean	84.5	93.0	84.7	90.0

**Table 3:** Rejection rates for target motions across sessions (A1–I1, A2–C2).

Session	BPNN-Entropy	OCSVM	OvR-RO
A1	8.6	26.3	0.0
A2	24.3	29.2	5.5
B1	24.1	29.3	9.2
B2	39.6	25.0	13.0
C1	40.9	25.0	14.3
C2	38.5	27.1	13.8
D1	33.3	26.2	7.1
E1	21.2	33.5	6.1
F1	30.0	25.6	13.8
G1	71.3	30.3	49.6
H1	41.7	34.3	36.9
I1	14.6	32.8	4.7
Mean	32.3	28.7	14.5

**Table 4:** Rejection precision for ambiguous motions across sessions (A1–I1, A2–C2). The symbol “–” indicates that no trials were rejected in that condition, so rejection precision was undefined.

Session	BPNN-Entropy	OCSVM	OvR-RO
A1	4.9	0.3	–
A2	30.8	6.4	45.7
B1	24.2	12.6	47.1
B2	39.5	11.4	42.5
C1	46.0	21.1	60.9
C2	49.8	9.1	46.8
D1	30.6	17.4	47.0
E1	42.2	10.6	54.4
F1	40.0	22.8	50.3
G1	51.8	44.5	62.9
H1	47.8	25.3	55.2
I1	39.9	12.6	65.2
Mean	37.3	16.2	52.5

**Table 5:** Rejection rates for unknown motions across sessions.

Session	BPNN-Entropy	OCSVM	OvR-RO
A2	91.0	98.0	67.0
B2	39.0	77.0	12.0
C2	42.0	36.0	26.0
F1	77.3	22.7	52.6
G1	84.0	37.0	69.0
H1	89.0	23.0	56.0
I1	79.3	20.7	16.1
Mean	71.7	44.9	42.7

**Table 6:** Rejection rates for non-target body movements (elbow flexion).

Session	BPNN-Entropy	OCSVM	OvR-RO
F1	16.0	98.0	0.0
G1	70.0	94.0	46.0
H1	92.0	2.0	56.0
I1	98.0	100.0	40.0
Mean	69.0	73.5	35.5

**Table 7:** Compact comparison of rejection mechanisms across motion categories. Abbreviations: Acc. = Accuracy, RR = Rejection Rate, RP = Rejection Precision, BPNN-E = BPNN-Entropy, Body Mov. = Body Movements, Ambig. = Ambiguous Motions (misclassified target-motion trials), OvR-RO = One-vs-Rest Rejection Option (proposed). Arrows indicate desirable direction (↑: higher is better, ↓: lower is better). Stars (★) denote best values per row. The row “Target (incl. Ambig.)” summarizes performance on the target-motion set, within which ambiguous trials are defined as misclassified target-motion trials.

Category	Metric	Baseline SVM	BPNN-E	OCSVM	OvR-RO
Target (incl. Ambig.)	Acc. [%] (↑)	84.5	${93.0\;}^{★}$	84.7	90.0
	RR [%] (↓)	—	32.3	28.7	${14.5\;}^{★}$
	RP [%] (↑)	—	37.3	16.2	${52.5\;}^{★}$
Unknown	RR [%] (↑)	0.0	${71.7\;}^{★}$	44.9	42.7
Body Mov.	RR [%] (↑)	0.0	69.0	${73.5\;}^{★}$	35.5

## Data Availability

The data presented in this study are available on request from the corresponding author. The data are not publicly available due to privacy and ethical restrictions.
